# Histone deacetylases in cancer metabolic reprogramming

**DOI:** 10.1038/s12276-026-01820-1

**Published:** 2026-09-02

**Authors:** Yu Hyun Jeon, Hyeyeong Seo, Ji Woo Youn, Jung Yoo, Sang Wu Lee, Soo Yeon Oh, So Hee Kwon

**Affiliations:** College of Pharmacy, Yonsei Institute of Pharmaceutical Sciences, Yonsei University, Incheon, 21983 Republic of Korea

**Keywords:** Cancer metabolism, Epigenetics

## Abstract

Cancer cells undergo extensive metabolic reprogramming to sustain rapid proliferation and adapt to heterogeneous tumour microenvironments. These metabolic alterations are tightly linked to epigenetic regulation, which reshapes gene expression and cellular signalling mechanisms. Among epigenetic regulators, histone deacetylases (HDACs) have emerged as key modulators of cancer metabolic reprogramming. Along with their canonical roles in histone deacetylation, HDACs regulate non-histone substrates, including metabolic enzymes and transcription factors, thereby coordinating transcriptional and metabolic programmes. In this review, we summarize current insights into HDAC-mediated regulation of glucose, lipid and amino acid metabolism in cancer and discuss the metabolic mechanisms underlying the anticancer effects of HDAC inhibitors. Collectively, we propose an integrated framework in which HDACs function as central regulators of cancer metabolic reprogramming. We highlight the limitations of HDAC inhibitor studies and discuss the emerging importance of isoform-specific HDAC functions in reprogramming cancer-specific metabolic dependencies and therapeutic strategies.

## Histone Deacetylases as Central Regulators of Cancer Metabolism

Histone deacetylases (HDACs) are crucial in epigenetic regulation, influencing gene expression and cellular metabolism. This review explores the roles of HDACs in cancer metabolic reprogramming, particularly in glucose, lipid and amino acid metabolism. The authors discuss how HDACs, through deacetylation, regulate both histone and non-histone proteins, impacting processes such as glycolysis, the Warburg effect and fatty-acid oxidation. They highlight significant findings, such as the role of HDAC1 in controlling glucose and lipid metabolisms and the regulation of glycolysis and glutamine metabolism in cancer by HDAC6. These insights underscore the potential of HDACs as therapeutic targets, with HDAC inhibitors already in clinical use for cancer treatment. The review suggests that developing isoform-specific HDAC inhibitors could enhance therapeutic precision, particularly in targeting cancer’s metabolic dependencies, pointing towards future research directions in cancer metabolism and epigenetics.

This summary was initially drafted using artificial intelligence, then revised and fact-checked by the author.

## Introduction

### Histone deacetylases: classification and function

**Fig. 1:**
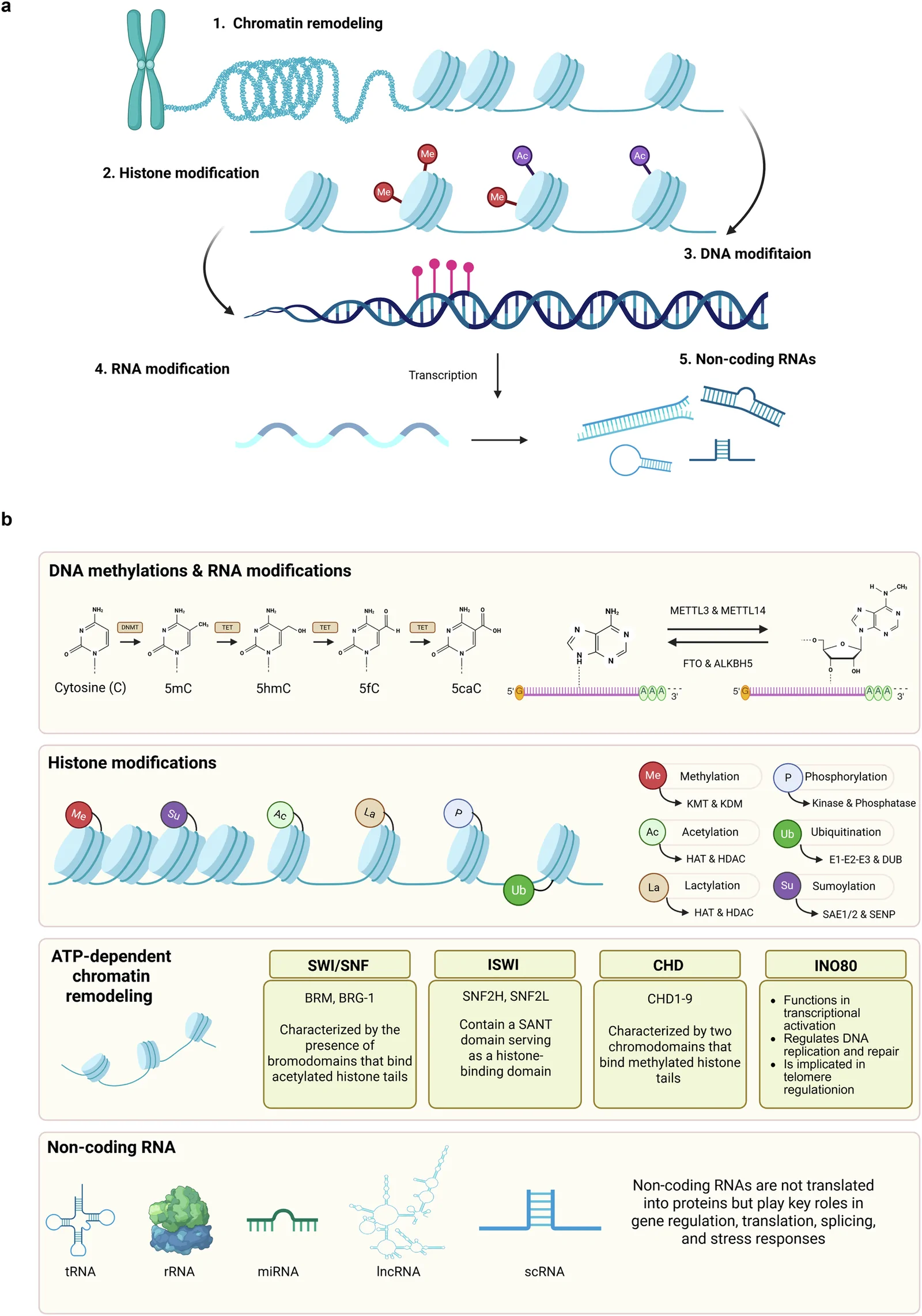
Overview of epigenetic regulation of gene expression. **a** The schematic summarizes major epigenetic regulatory layers, including DNA methylation, RNA modifications, histone modifications, chromatin remodelling, and non-coding RNAs. These mechanisms cooperatively regulate chromatin accessibility and transcriptional outcomes without altering the DNA sequence. **b** DNA methylation and demethylation are one of the most prevalent epigenetic modifications, in which DNA methyltransferase (DNMTs) add methyl groups to cytosine residues, whereas ten-eleven translocation (TET) enzymes mediate active demethylation through oxidation of 5-methylcytosine. DNA methylation predominantly occurs at promoter regions and plays a critical role in transcriptional regulation. RNA methylation, particularly N6-methyladenosine (m6A), represents the most abundant internal mRNA modification and is catalysed by the methyltransferase-like 3 (METTL3)–methyltransferase-like 14 (METTL14) complex, with Fe(II)/α-ketoglutarate-dependent dioxygenase (FTO) and AlkB homolog 5, RNA demethylase (ALKBH5) acting as demethylase. Histone modifications, including methylation, acetylation, lactylation, phosphorylation, ubiquitination and sumoylation, occur mainly on histone N-terminal tails and dynamically regulate chromatin structure. Chromatin accessibility is further controlled by ATP-dependent nucleosome remodelling complexes, including the SWItch/Sucrose Non-Fermentable (SWI/SNF), Imitation SWItch (ISWI), Chromodomain Helicase DNA-binding (CHD), and Inositol requiring 80 (INO80) families. In addition, non-coding RNAs, comprising short and long non-coding RNAs, contribute to epigenetic regulation by modulating transcription and RNA metabolism. lncRNA, long non-coding RNA; miRNA, microRNA; rRNA, ribosomal RNA; scRNA, single-cell RNA; tRNA, transfer RNA.

Epigenetic regulation involves DNA modifications, RNA modifications, histone modifications, chromatin remodelling and non-coding RNAs, all of which reversibly regulate gene expression^1^ (Fig. 1). Consequently, epigenetic modifications profoundly influence cellular metabolism and are tightly linked to metabolic regulation. Among post-translational modifications, deacetylation plays a crucial role in regulating diverse cellular processes^2^. Histone acetylation and deacetylation are dynamic and reversible epigenetic modifications that modulate chromatin structure and transcriptional activity^3^. Canonically, histone deacetylases (HDACs) remove acetyl groups from ε-lysine residues on histone tails, promoting chromatin compaction and transcriptional repression at target genomic loci. In this review, we focus on HDACs and their emerging roles in cancer metabolic reprogramming.

**Fig. 2:**
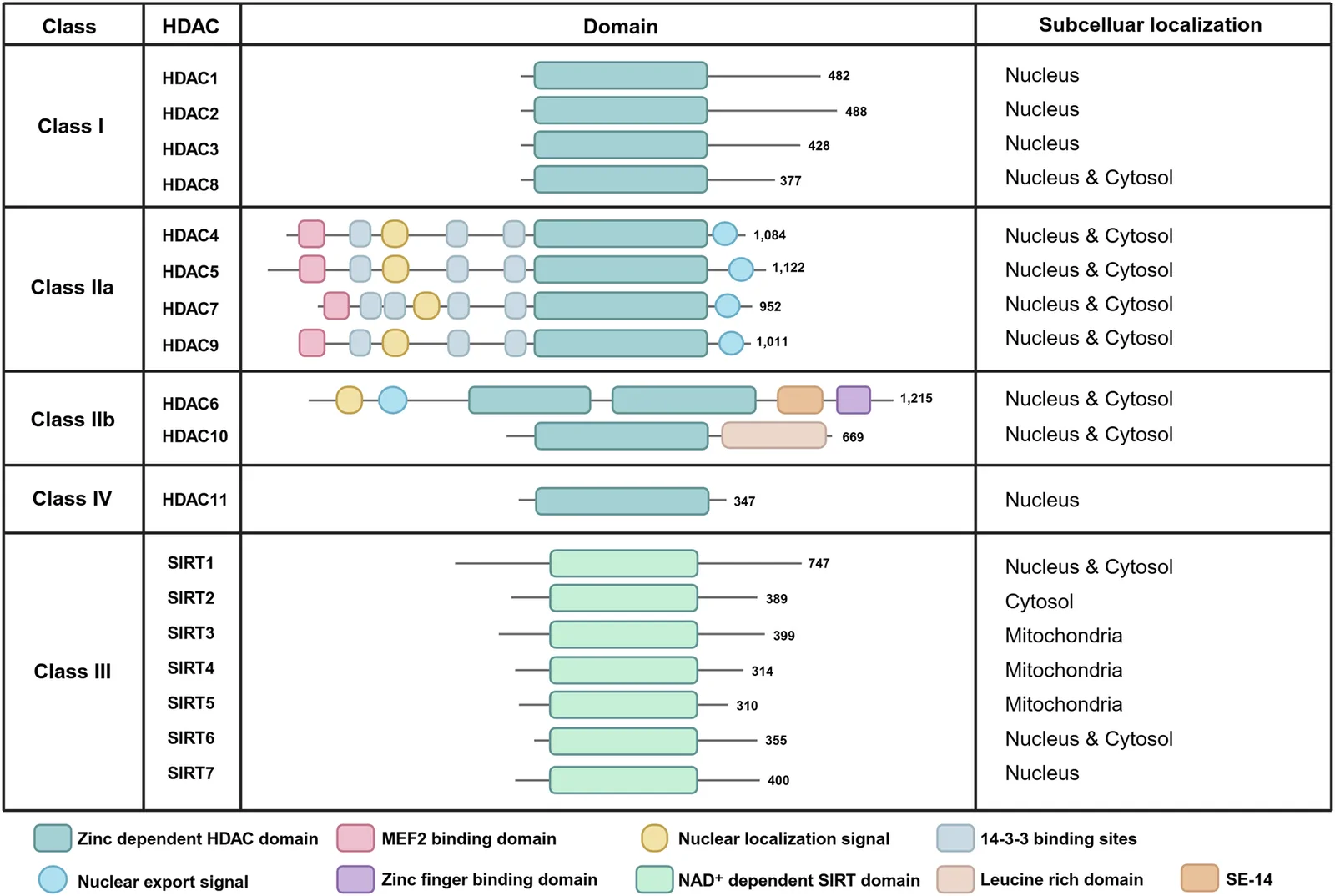
Classification and structural organization of the histone deacetylase family. Histone deacetylase (HDAC) family members are grouped into class I, class IIa, class IIb, class III and class IV, with a schematic representation of their conserved catalytic domains and associated regulatory regions. Differences in structural features among HDAC isoforms contribute to their functional roles and subcellular localization.

HDACs function not only in chromatin regulation but also in deacetylate diverse non-histone proteins^4^, including transcription factors^5^ and metabolic enzymes^6^, a property central to their biological and pathological functions. Through these activities, HDACs act as integrative regulators that couple epigenetic control to signal transduction^7^, metabolism^8^ and cell-cycle progression^9^. This functional diversity is reflected in the structural and regulatory heterogeneity of the HDAC family. The mammalian HDAC family includes 18 isoforms, which are classified into four classes (I–IV); among these, the Zn^2+^-dependent classes I, II and IV are the focus of this review^10^ (Fig. 2). Class I HDACs are predominantly nuclear enzymes with robust deacetylase activity and primarily function within large multiprotein corepressor complexes to enforce transcriptional repression and chromatin stability^11^. In contrast, class II HDACs shuttle between cellular compartments in a signal-dependent manner and act as regulatory scaffolds, linking transcriptional regulation to cytoplasmic processes^12^. These functions underscore the expanding role of HDACs that are not limited to chromatin regulation and highlight their contribution to cellular homeostasis at multiple regulatory levels. HDAC11, the only class IV histone deacetylase, shares structural features with both class I and class II enzymes and has been implicated in immune response^13^ and metabolic signalling^14^. Collectively, these class-specific properties underscore how HDACs regulate cellular homeostasis across multiple layers, from chromatin remodelling to non-histone protein modifications.

Despite shared structural features and overlapping substrates, individual HDAC isoforms exhibit distinct and non-redundant functions, including opposing roles in cancer. Such isoform-specific distinctions are particularly evident in cancer metabolism, where individual HDACs play opposing roles. For example, class I HDACs, particularly HDAC8, promote glycolysis to drive hepatocellular carcinoma (HCC) proliferation^15^. In contrast, class IIa HDACs function as tumour suppressors by maintaining mitochondrial homeostasis, as exemplified by HDAC5, whose loss triggers metabolic adaptation and ultimately autophagy and apoptosis. Also, class IIb HDAC6 promotes tumour progression by stabilizing glutamine synthetase and facilitating glutamine metabolism in glioblastoma (GBM)^16^. Therefore, a precise understanding of individual HDAC isoform functions is essential for identifying their non-redundant roles in cancer metabolism.

Given their central role in epigenetic regulation, HDACs have emerged as attractive therapeutic targets, leading to the clinical development of HDAC inhibitors^17^. The clinical relevance of HDAC inhibition has been validated by the regulatory approval of several HDAC inhibitors, including vorinostat, romidepsin, panobinostat and belinostat, for the treatment of cancer^18^. Nevertheless, the therapeutic efficacy of HDAC inhibitors remains limited in many cancer types, particularly in solid tumours and those associated with metabolic dysregulation. Emerging evidence indicates that HDACs regulate key metabolic enzymes and pathways, suggesting that metabolic reprogramming may underlie resistance to HDAC inhibitor therapy. In this review, we focus on the class-specific mechanisms by which HDACs integrate epigenetic and metabolic signalling inputs and highlight their roles in disease pathogenesis and therapeutic targeting.

## Metabolism in cancer

### Metabolic reprogramming in cancer

Metabolic reprogramming is a hallmark of cancer^3^, enabling tumour cells to adapt their energy production and biosynthetic processes to support rapid growth, survival and metastasis. Beyond the classical Warburg effect, defined as a shift towards aerobic glycolysis, cancer cells dynamically remodel multiple metabolic networks, including glucose, lipid and amino acid metabolism, to sustain proliferative capacity and stress resistance^6^.

Within this framework, glucose metabolism is frequently redirected towards aerobic glycolysis and the pentose phosphate pathway (PPP), ensuring a continuous supply of ATP, NADPH and nucleotide precursors required for anabolic growth^19^. Notably, this metabolic shift does not imply impaired mitochondrial function. Instead, mitochondrial metabolism remains active, with the tricarboxylic acid (TCA) cycle and oxidative phosphorylation (OXPHOS) supporting biosynthesis and redox homeostasis^20^. In parallel, glutamine acts as a major carbon and nitrogen source, replenishing TCA cycle intermediates and fuelling lipid synthesis through glutaminolysis^21^. Consistent with this coordinated metabolic remodelling, lipid metabolism is extensively reprogrammed, as enhanced fatty-acid synthesis and β-oxidation contribute to membrane biogenesis, energy production and redox control. Together, these interconnected pathways underscore the metabolic plasticity of cancer cells, enabling dynamic adaptation to hypoxia, nutrient availability, and oncogenic signalling.

Emerging evidence indicates that these metabolic adaptations are tightly coordinated with epigenetic regulation, including histone modifications and chromatin remodelling, thereby defining transcriptional states associated with altered metabolic pathways in cancer^2^. Central metabolites such as S-adenosylmethionine (SAM) and acetyl-CoA function not only as key metabolic intermediates but also as indispensable methyl and acetyl donors for the histone modification process^22^. Thus, metabolic reprogramming constitutes an interconnected and highly adaptable process that interfaces with epigenetic mechanisms to promote tumour growth, survival and disease progression.

### Warburg effect

**Fig. 3:**
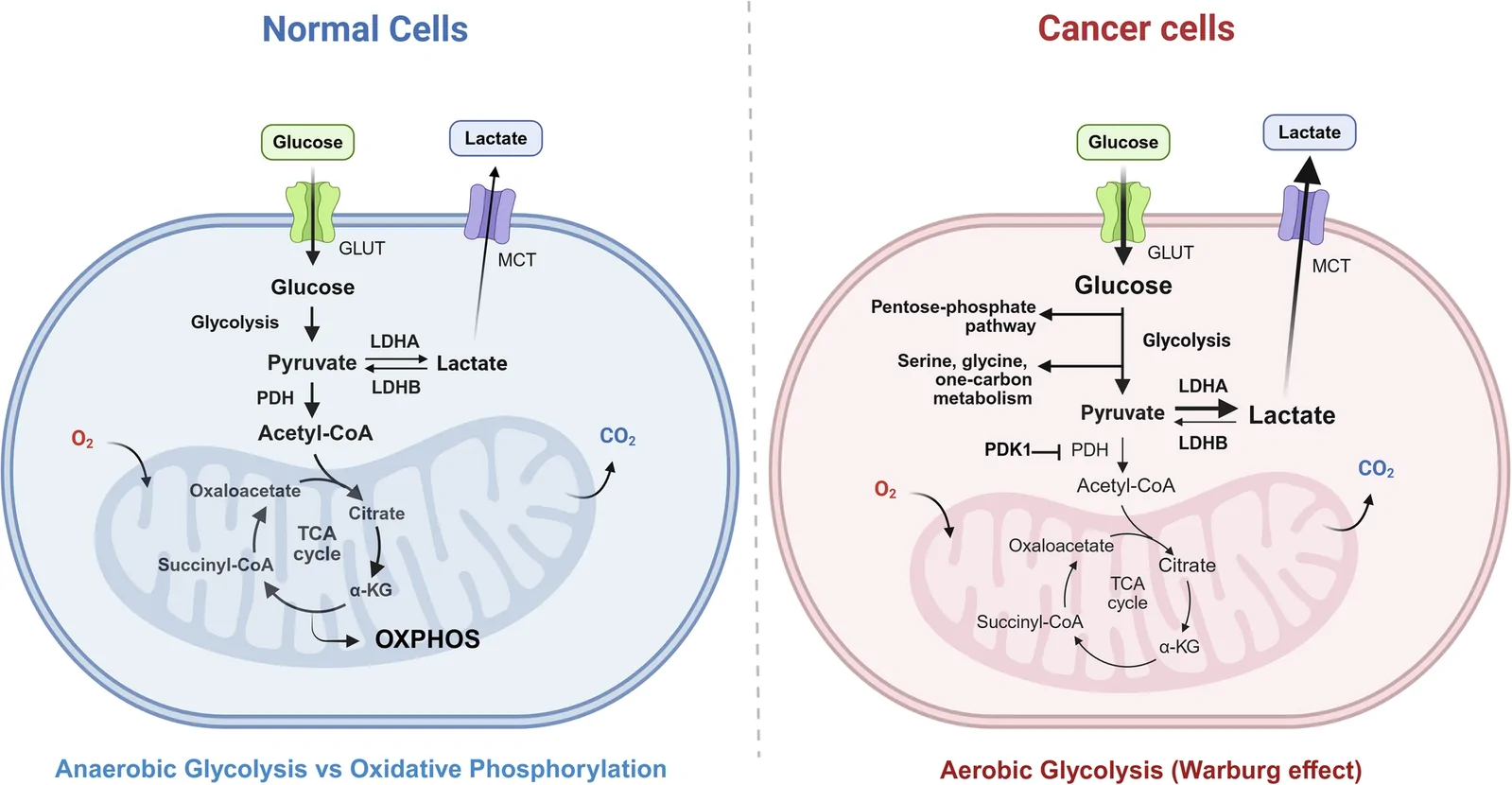
Anaerobic glycolysis in normal and cancer cells. Normal cells mainly rely on mitochondrial oxidative phosphorylation (OXPHOS), in which glucose-derived pyruvate enters the tricarboxylic acid (TCA) cycle via pyruvate dehydrogenase (PDH) to efficiently generate ATP. In contrast, cancer cells preferentially exhibit the Warburg effect, characterized by increased glucose uptake and aerobic glycolysis with lactate production, even under normoxic conditions. Although less efficient for ATP yield, glycolysis supports rapid energy production and provides biosynthetic intermediates that promote tumour growth.

The Warburg effect describes a characteristic metabolic feature of cancer metabolism in which tumour cells preferentially rely on aerobic glycolysis, converting glucose to lactate despite adequate oxygen availability^23^ (Fig. 3). Although mitochondrial OXPHOS is more efficient in terms of ATP yield, glycolysis enables rapid ATP production while supplying abundant metabolic intermediates that fuel the biosynthesis of nucleotides, amino acids and lipids required for sustained proliferation^24,25^.

This metabolic shift is driven by the activation of oncogenic pathways and transcriptional regulators that collectively rewire cellular metabolism. Upregulation of glucose transporters (GLUT1 and GLUT3)^26^ and key glycolytic enzymes such as hexokinase 2 (HK2)^27^ and PKM2^28^ enhances glycolytic flux and lactate production. Oncogenes such as Myc^29^ and stabilized HIF-1α^30^ under hypoxia promote the transcription of glycolytic genes, whereas tumour suppressors, including p53^31^ and LKB1^32^, normally counterbalance these effects by maintaining mitochondrial respiration and AMPK activity.

Apart from energy production, the Warburg effect provides several metabolic advantages for cancer cells. The diversion of glycolytic intermediates into PPP generates NADPH for redox balance and lipid synthesis, and ribose-5-phosphate for nucleotide biosynthesis^33^. Accumulation of lactate also contributes to the acidification of the tumour microenvironment, which facilitates immune evasion and invasion^34^.

## Carbohydrate and glucose metabolism

Glucose metabolism is profoundly reprogrammed in cancer cells to support rapid proliferation through elevated glucose uptake and its preferential conversion to lactate, even under aerobic conditions. This metabolic rewiring provides essential intermediates for lipid, nucleotide, and protein biosynthesis, while sustaining cellular energy demands^6^. Importantly, these metabolic alterations are tightly regulated by epigenetic modifications^2^. Thus, dysregulation of glucose metabolism through epigenetic regulation represents a central feature of cancer metabolism.

### Histone deacetylase regulation of the Warburg effect

**Fig. 4:**
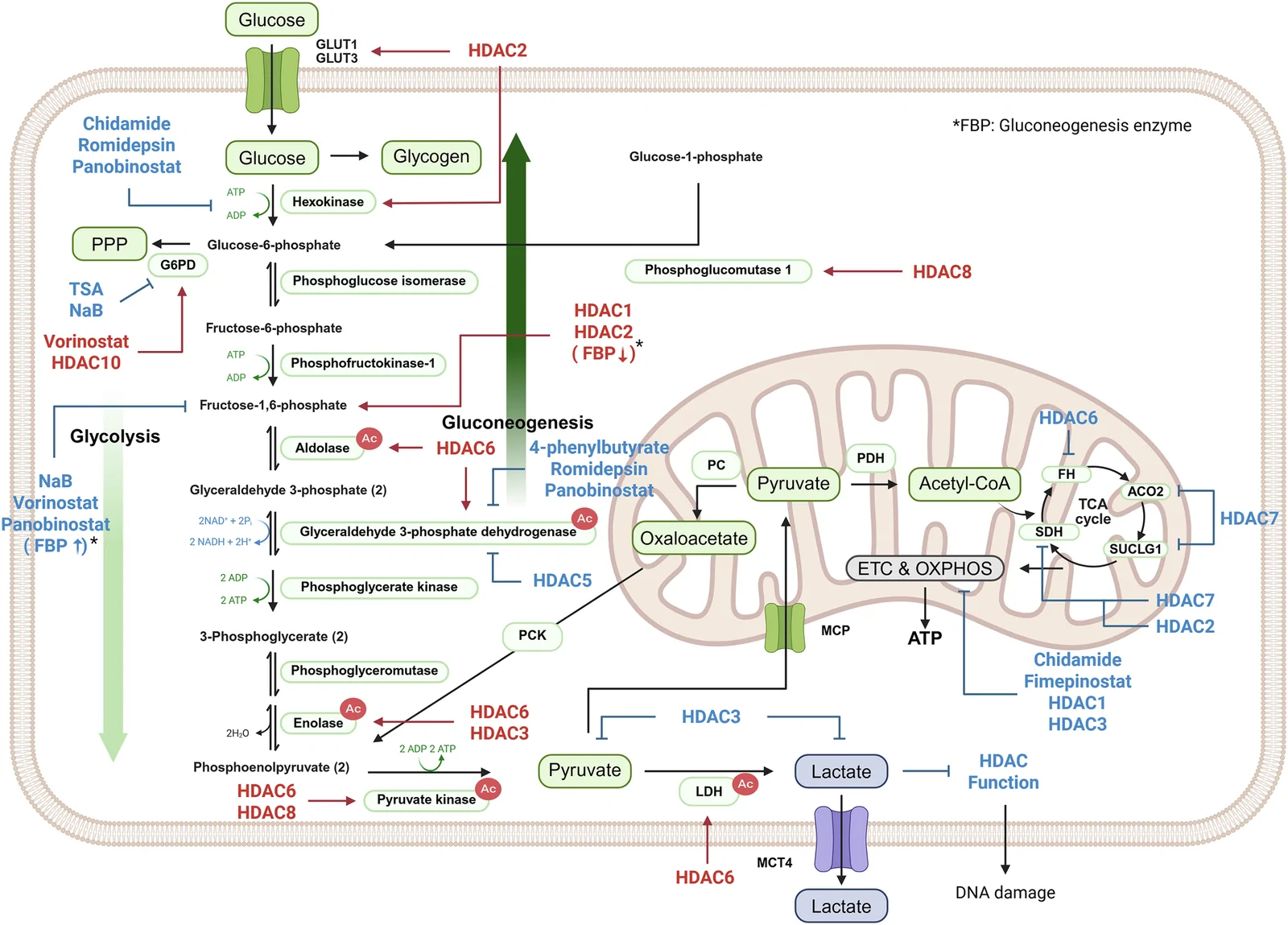
Histone deacetylases and histone deacetylase inhibitors affect carbohydrate metabolism in cancer. Glucose uptake via glucose transporters (GLUT) fuels glycolysis, glycogen metabolism and pentose phosphate pathway (PPP), with distinct histone deacetylase (HDAC) isoforms positioned at key control points. HDAC1 and HDAC2 are associated with glucose entry and early glycolytic regulation, whereas HDAC8 is linked to PGM1 and glucose-1-phosphate metabolism. Pyruvate metabolism and mitochondrial oxidative phosphorylation (OXPHOS) link cytosolic pathways to mitochondrial energy production. HDAC inhibitors (e.g. chidamide, romidepsin, panobinostat and 4-phenylbutyrate), alone or in combination with other agents, disrupt metabolic flux and induce metabolic stress. Glucose-derived glucose-6-phosphate enters the oxidative PPP through G6PD, generating NADPH. Vorinostat, trichostatin A (TSA), and NaB are placed near the PPP entry step to highlight pharmacological modulation of NADPH-producing pathways. HDAC inhibition regulates PPP-derived NADPH and limits metabolic flexibility required for proliferation.

In tumours, glucose uptake is markedly increased, and lactate is produced despite sufficient oxygen and intact mitochondria, a phenomenon known as the Warburg effect^23^. Beyond serving as a metabolic consequence of oncogenic signalling, the Warburg effect is actively regulated by epigenetic mechanisms, including HDAC-dependent transcriptional control of glycolytic genes. Butyrate exemplifies this bidirectional relationship between metabolism and epigenetic control. In normal cells, butyrate is readily oxidized and serves as an energy source. In contrast, in cancer cells with high glycolytic activity, butyrate oxidation is reduced, leading to intracellular accumulation and HDAC inhibition^35^. A similar principle applies to lactate, a key metabolic product of glycolysis that accumulates under Warburg conditions. In another study, HDAC inhibitors were employed as functional mimetics of lactate, suggesting that lactate-driven transcriptional changes occur through HDAC inhibition^36^. Elevated lactate suppresses HDAC activity, thereby promoting expression of DNA damage repair proteins, which ultimately enhances cell survival and contributes to drug resistance in cervical cancer. Supporting this notion, the Warburg effect is enhanced by super enhancer-driven expression of metabolic genes, including HK2 and solute carrier family 2 member 1 (SLC2A1)^37^. These super enhancer regions are enriched for H3K27ac and co-localize with HDAC2. Pharmacological inhibition of HDAC2 with romidepsin or panobinostat disrupts these regions, resulting in reduced Myc, HK2, and glyceraldehyde 3-phosphate dehydrogenase (GAPDH) expression at both mRNA and protein (Tables 1 and 2, Fig. 4).

**Table 1:** Regulation of metabolic enzymes by histone deacetylase isoforms.

	HDAC	Metabolic target	Pathway	Regulation	Refs.
Class I	HDAC1	FBP1	Gluconeogenesis	Down	^76^
		CPT1	FAO	Up	^83^
	HDAC2	GLUT3	Glycolysis	Up	^55^
		HK2	Glycolysis	Up	^37^
		FBP1	Gluconeogenesis	Down	^76^
		SDHA	TCA	Down	^65^
	HDAC3	FASN	FAS	Down	^87^
		ENO2	Glycolysis	Up	^56^
	HDAC8	PGM1	Glycolysis	Up	^58^
		PKM2	Glycolysis	Up	^15^
Class IIa	HDAC4	GLS	Glutamine metabolism	Up	^95^
	HDAC5	GAPDH	Glycolysis	Up	^50^
		FASN	FAS	Up	^89^
	HDAC7	ACO2	TCA	Down	^66^
		SUCLG1	TCA	Down	
		SDHC	TCA	Down	
Class IIb	HDAC6	GS	Glutamine metabolism	Up	^16^
		Aldolase	Glycolysis	Up	^59^
		GAPDH	Glycolysis	Up	
		Enolase	Glycolysis	Up	
		LDHA	Lactate metabolism	Up	
	HDAC10	G6PD	PPP	Up	^63^

Upregulation and downregulation indicate increased or decreased expression or activity of the indicated metabolic enzyme upon modulation of the corresponding HDAC isoform. *FAO* fatty-acid oxidation, *FAS* fatty-acid synthase, *HDAC* histone deacetylase, *PPP* pentose phosphate pathway, *TCA* tricarboxylic acid.

**Table 2:** Regulation of metabolic enzymes by histone deacetylase inhibitors.

	Drug	Metabolic target	Pathway	Regulation	Refs.
Pan-HDAC	Trichostatin A	G6PD	PPP	Down	^63^
		Ribose-5-phosphate	PPP	Down	^63^
	Panobinostat	HK2	Glycolysis	Down	^52^
		GAPDH	Glycolysis	Down	^37^
		FBP1	Gluconeogenesis	Up	^76^
	4-phenylbutyrate	GAPDH	Glycolysis	Down	^49^
	Vorinostat	FBP1	Gluconeogenesis	Up	^76^
		G6PD	PPP	Up	^62^
	NaB	FBP1	Gluconeogenesis	Up	^76^
		Ribose-5-phosphate	PPP	Down	^61^
Class I	Romidepsin	HK2	Glycolysis	Down	^37^
		GAPDH	Glycolysis	Down	^37^
	Chidamide	HK1	Glycolysis	Down	^71^
HDAC6	Citarinostat	GS	Glutamine metabolism	Down	^16^
	Tubastatin A	GS		Down	
	A452	GS		Down	
HDAC & PI3K	Fimepinostat	OXPHOS	OXPHOS	Down	^72^

Upregulation and downregulation refer to increases or decreases in the expression or activity of the indicated metabolic enzyme following treatment with the corresponding HDAC inhibitor. *HDAC* histone deacetylase, *OXPHOS* oxidative phosphorylation, *PPP* pentose phosphate pathway.

The Warburg effect induces lactate production and supports energy for cell proliferation. Therefore, lactate dehydrogenase A (LDHA) has increased in various cancers, resulting in a low pyruvate level^8^. Reduced pyruvate availability protects cholangiocarcinoma cells from apoptosis. In parallel, HDAC3 stabilizes c-Myc through deacetylation at K323, thereby reinforcing glycolytic metabolism and sustaining a low pyruvate state. This effect may be supported by enhanced pyruvate-to-lactate conversion, forming a positive feedback loop that promotes tumour cell survival^38^. Consistently, HDAC6-mediated deacetylation of transaldolase 1 (TALDO1) promotes its degradation, leading to increased expression of glycolytic enzymes, including LDHA, HK and PDK1 in nasopharyngeal carcinoma^8^. Together, these findings indicate that HDACs act as key mediators linking glycolytic metabolism to epigenetic regulation, thereby sustaining the Warburg phenotype.

### Histone deacetylase-mediated control of lactylation

Lactate was initially regarded as a byproduct of glycolysis but is now recognized as a metabolite associated with lysine lactylation. In tumours, lactate accumulation promotes enhanced lactylation, extending the relevance of the Warburg effect beyond energy metabolism and contributing to tumorigenesis and therapeutic resistance^39–41^. Recent studies indicate that enzymes classically defined as histone acetyltransferase (HAT) and HDACs can also catalyse lactylation and delactylation: CBP and p300 as lactyl writers and HDAC1-3, SIRT1-3 and SIRT6 as lactyl erasers^42,43^. In triple-negative breast cancer (TNBC), HDAC2-mediated delactylation of methyltransferase-like 3 (METTL3) enhances DNA damage repair and contributes to the development of chemotherapy resistance, whereas pharmacological inhibition of HDAC2 using tucidinostat increases sensitivity to cisplatin^44^. In addition, lactylation of Nijmegen breakage syndrome 1 (NBS1) is associated with increased Mre11-Rad50-Nbs1 (MRN) complex assembly at DNA double-strand breaks, thereby facilitating DNA repair and resistance to DNA-damaging chemotherapy^45^. HDAC3 has been reported to mediate the delactylation of NBS1. Increased NBS1 lactylation in tumours shows an association with reduced HDAC3 activity and unfavourable clinical outcomes. Along these lines, HDAC3-mediated delactylation restrains RHOA activation and promotes its degradation^46^. These findings indicate that HDAC3 is involved in the regulation of lactylation-associated oncogenic signalling. Collectively, these studies indicate that lactate accumulation by reprogramming metabolism induces lactylation of non-histone proteins, whereas HDACs function as delactylases to modulate oncogenic signalling pathways and influence tumour progression.

### Histone deacetylase-mediated regulation of glycolysis

Enhanced glycolysis promotes cancer-cell survival and proliferation by increasing glucose uptake and metabolic flux. Accordingly, glycolytic enzymes and metabolites are frequently upregulated in cancer cells, a process that is tightly regulated by oncogenic transcription factors and epigenetic modifications^47^.

In this context, GAPDH is an enzyme involved in the process of glycolysis^48^. GAPDH catalyses phosphorylation and oxidation of glyceraldehyde-3-phosphate to 1,3-biphosphoglycerate, using NAD^+^ as the electron acceptor. In glioma cells, HDAC inhibitor 4-phenylbutyrate (4-PB) inhibits cell proliferation and induces apoptosis by decreasing GAPDH mRNA level^49^. GAPDH activity is positively regulated by acetylation at lysine 254 (K254), which is stimulated by glucose availability^50^. The acetyltransferase PCAF promotes K254 acetylation and enhances GAPDH enzymatic activity, whereas HDAC5 deacetylates K254 and reduces its activity. This reversible acetylation cycle allows cells to dynamically tune glycolytic flux in response to extracellular glucose levels. These findings collectively suggest that HDAC-mediated regulation of GAPDH contributes to glycolytic flux in cancer cells. Other key glycolytic enzymes, including hexokinase 2 (HK2), are also subject to HDAC-dependent regulation. HK2 is another key regulator of glucose metabolism that converts glucose to glucose 6–phosphate^51^. HK2 is frequently overexpressed in cancer and has been associated with chemoresistance. Diffuse large B cell lymphoma (DLBCL) is a metabolically heterogeneous disease that can be broadly classified into glycolysis-dependent and OXPHOS-dependent subtypes. In DLBCL patients, HK2 is highly expressed through the activation of both c-Myc and HIF1. Panobinostat suppresses HK2 expression under both normoxia and hypoxia, thereby overcoming cisplatin resistance through inhibition of glycolysis^52^. In contrast, OXPHOS-DLBCL cells exhibit intrinsic resistance to pan-HDAC inhibitors such as pracinostat and vorinostat, whereas BCR-dependent DLBCL cells show marked sensitivity to pracinostat^53^. These findings suggest that HDAC inhibition is more effective in lymphoma subtypes characterized by heightened glycolytic dependency.

Supporting this mechanism, specific HDACs have been shown to drive cancer progression by directly regulating tumour metabolic programmes. HDAC1 is implicated in cancer progression through metabolic regulation. In endometrial cancer, HDAC1 is upregulated and correlates with advanced clinicopathological progression through enhanced glucose consumption and lactate secretion^22^. Similarly, HDAC8, another class I member, deacetylates PKM2 at K62, promoting its nuclear localization and glycolysis to drive HCC proliferation^15^. Moreover, HDAC-dependent epigenetic regulation plays a critical role in glycolytic control by suppressing the expression of SIRT6, a known metabolic gatekeeper^54^. Mechanistically, HDAC1-mediated deacetylation of E2F1 facilitates its function as a transcriptional repressor of the SIRT6 promoter, thereby enhancing glycolysis in cancer cells. Another class I HDAC, HDAC2, is highly expressed in GBM, where it promotes tumour progression by regulating glucose metabolism^55^. HDAC2 suppresses miR-3189, leading to sustained expression of the glucose transporter GLUT3. Inhibition of GLUT3 results in reduced glucose uptake, attenuated glycolytic activity and impaired cellular proliferation. These findings indicate that HDAC2 drives glucose uptake through GLUT3, thereby linking metabolic reprogramming to GBM progression. Pharmacological inhibition of HDAC activity using trichostatin A (TSA) increases E2F1 acetylation, leading to reactivation of SIRT6 expression and attenuation of glycolytic flux.

Beyond glucose uptake, HDACs directly regulate glycolytic enzyme activity. Enolase 2 (ENO2) is a key glycolytic enzyme whose activity is regulated by HDAC3 in pancreatic ductal adenocarcinoma^56^. HDAC3-mediated deacetylation of ENO2 at K394 enhances glycolysis in response to insulin-like growth factor 1. These findings demonstrate that HDACs directly regulate glycolytic enzymes to promote metabolic reprogramming in cancer. Similar observations have been reported in HeLa cells, where TSA suppresses glycolysis in association with altered regulation of HIF1α and c-Myc^57^. Recent studies provide evidence that metabolic stress conditions critically shape HDAC-dependent metabolic regulation. Specifically, under glucose starvation, AMPK-dependent phosphorylation of HDAC8 promotes phosphoglucomutase 1 (PGM1) expression, enabling metabolic flexibility and supporting survival of lung-cancer cells^58^. In addition, class IIb HDAC6 has emerged as a major regulator of glycolytic metabolism in TNBC. Inhibition of HDAC6 leads to reduced glucose uptake and lactate production, resulting in impaired glycolytic flux. Mechanistically, HDAC6 interacts with multiple glycolytic enzymes, including aldolase, GAPDH, enolase and LDHA. Inhibition of HDAC6 increases the acetylation of these enzymes, which is associated with impaired glycolytic activity^59^. Furthermore, sorafenib-induced upregulation of HDAC6 activity enhances PKM2 expression and glycolytic flux, a mechanism implicated in the development of sorafenib resistance in HCC^60^. Collectively, HDACs regulate glycolysis through two non-mutually exclusive mechanisms that include transcriptional activation of glycolytic genes and post-translational modulation of glycolytic enzymes (Fig. 4).

### Histone deacetylase-driven regulation of the pentose phosphate pathway

The PPP, which branches out from glycolysis, is essential for ribonucleotide synthesis and serves as a major source of NADPH. In cancer cells, PPP activity supports fatty-acid and nucleotide biosynthesis and contributes to redox homeostasis by reducing reactive oxygen species, thereby promoting cell survival.

Treatment with pan-HDAC inhibitors TSA or sodium butyrate (NaB) in lung cancer reduces glycolytic flux and decreases ribose-5-phosphate levels, indicating diminished utilization of the PPP^61^. The reduced proportion observed after HDAC inhibition reflects attenuated PPP flux accompanying cellular differentiation. In contrast, HDAC inhibitor treatment paradoxically induces cancer stem-cell enrichment in TNBC. Debeb et al. reported that valproic acid or vorinostat increases ALDH^+^ cancer stem cells accompanied by PPP activation via glucose-6-phosphate dehydrogenase (G6PD) upregulation and NADPH production^62^. Beyond the effects of HDAC inhibition, HDAC10 plays a direct role in PPP regulation. In lung cancer, HDAC10 promotes tumour growth by upregulating G6PD, thereby enhancing PPP flux to support biosynthetic demands of rapidly proliferating cancer cells^63^. This metabolic reprogramming enhances redox balance, thereby promoting cell survival and drug resistance, despite HDAC inhibitors inducing cytotoxicity. Collectively, these findings indicate that the impact of HDAC inhibition on PPP activity is highly context dependent and can differentially influence cancer-cell survival under conditions of metabolic and oxidative stress. Additional studies are required to determine how this modulation influences cancer-cell survival and to inform therapeutic strategies (Tables 1 and 2).

### Histone deacetylase control of the tricarboxylic acid cycle and oxidative phosphorylation

The TCA cycle serves as a central metabolic pathway that integrates carbon metabolism with mitochondrial respiration by supplying reducing equivalents to the electron transport chain. Some intermediates of the TCA cycle are dysregulated in cancer and are associated with tumour progression^64^. Succinate dehydrogenase subunit A (SDHA) promotes the TCA cycle and suppresses glycolysis. In multiple myeloma, low expression of endothelial PAS domain protein 1 (EPAS1) induces HDAC2 expression, which represses SDHA transcription and enhances glycolytic metabolism^65^. Similarly, in renal-cell carcinoma (RCC), reduced expression of the TCA-cycle enzyme is related to poor prognosis. The TGFβ–HDAC7 axis suppresses multiple TCA-cycle enzymes, including aconitase 2 (ACO2), Succinate-CoA ligase alpha subunit (SUCLG1) and SDHC, whereas inhibition of HDAC7 restores TCA-cycle metabolism in RCC^66^. Additionally, HDAC6 inhibition disrupts mitochondrial metabolism by impairing fumarate hydratase activity and mitochondrial architecture, thereby linking epigenetic regulation to TCA-cycle integrity and mitochondrial function^67^.

Cancer cells typically exhibit increased glycolysis compared with normal cells, a phenomenon historically associated with reduced reliance on OXPHOS. However, accumulating evidence indicates that OXPHOS is not universally impaired in cancers^68^. Instead, several malignancies display enhanced mitochondrial respiration with elevated OXPHOS activity. Upregulated OXPHOS has been associated with poor prognosis, drug resistance and cancer stemness, as reported in leukaemia and pancreatic cancer^69,70^. Accordingly, targeting OXPHOS has been a novel therapeutic strategy for OXPHOS-high cancer, improving treatment outcomes by reducing tumour hypoxia. Acute myeloid leukaemia (AML) represents a prototypical OXPHOS-dependent malignancy. High expression of HDAC1 and HDAC3 is significantly associated with poor clinical outcomes in patients with AML^71^. Pharmacological inhibition of HDAC1 and HDAC3 by chidamide suppresses AML-cell proliferation and survival by disrupting OXPHOS through downregulation of HK1 and mitochondrial ATP production. These findings highlight a critical role for HDAC1 and HDAC3 in maintaining OXPHOS activity. Consistently, fimepinostat (CUDC-907), a dual inhibitor of PI3K and HDAC, has been shown to synergize with venetoclax, a selective BCL2 inhibitor, in AML by suppressing OXPHOS through inhibition of c-Myc and BCL2 signalling^72^. Together, these studies suggest that HDACs regulate the TCA cycle and OXPHOS in cancer, supporting their potential as therapeutic targets in OXPHOS-dependent malignancies (Tables 1 and 2).

### Histone deacetylases in gluconeogenesis and glycogen metabolism

Gluconeogenesis generates glucose from non-carbohydrate carbon sources, such as lactate and amino acids, and is tightly regulated by rate-limiting enzymatic steps distinct from glycolysis^73^. Although phosphoenolpyruvate carboxykinase (PEPCK)-dependent gluconeogenic reactions can be rewired to support anabolic metabolism in cancer^74^, fructose-1,6-bisphosphatase 1 (FBP1) constrains glycolytic flux, and its loss is associated with enhanced glycolysis and poor prognosis^75^. Epigenetic regulation has emerged as a key mechanism underlying FBP1 suppression in cancer. HDAC1 and HDAC2 repress FBP1 expression through the regulation of H3K27 acetylation at its enhancer region in HCC^76^. Consistently, treatment with HDAC inhibitors, including NaB, vorinostat and panobinostat, restores FBP1 expression at both the mRNA and protein levels in HCC cells^76^. These findings suggest that HDAC-mediated repression of gluconeogenic enzymes such as FBP1 represents an indirect but highly effective strategy for enforcing glycolytic dependency in cancer cells. The class I HDAC inhibitor Ky-2 significantly reduces PEPCK expression by suppressing the activity of hepatocyte nuclear factor 4α (HNF4α) and forkhead box O1 (FOXO1) in HepG2 cells^77^. Notably, among class I HDACs, only HDAC1 knockdown recapitulates the reduction in PEPCK expression, indicating a dominant role for HDAC1 in the regulation of gluconeogenesis. Consequently, inhibition of class I HDAC activity contributes to improved glucose metabolism homeostasis (Tables 1 and 2).

## Lipid metabolism

Lipid metabolism is extensively reprogrammed in cancer, characterized by the upregulation of lipid uptake, de novo lipid synthesis, storage and utilization to support rapid cell growth and promote tumour progression^78^. Notably, these metabolic programmes are closely intertwined with epigenetic regulation, as histone acetylation and methylation are influenced by metabolic intermediates such as acetyl-CoA and coordinated by lipid-associated transcription factors, including sterol regulatory element binding proteins (SREBPs)^22,79^.

### Histone deacetylase regulation of fatty-acid oxidation

Fatty acid oxidation (FAO) is a central metabolic pathway that converts fatty acids into acetyl-CoA, NADH and FADH_2_, providing substrates for the TCA cycle and OXPHOS^80^. The expression of FAO-related genes, including CD36, carnitine palmitoyltransferase 1 (CPT1) and CPT2, is overexpressed in various cancers^22,81,82^. Also, metabolites such as acetyl-CoA, NAD^+^ and AMP can modulate FAO through enzyme regulation and epigenetic mechanisms.

**Fig. 5:**
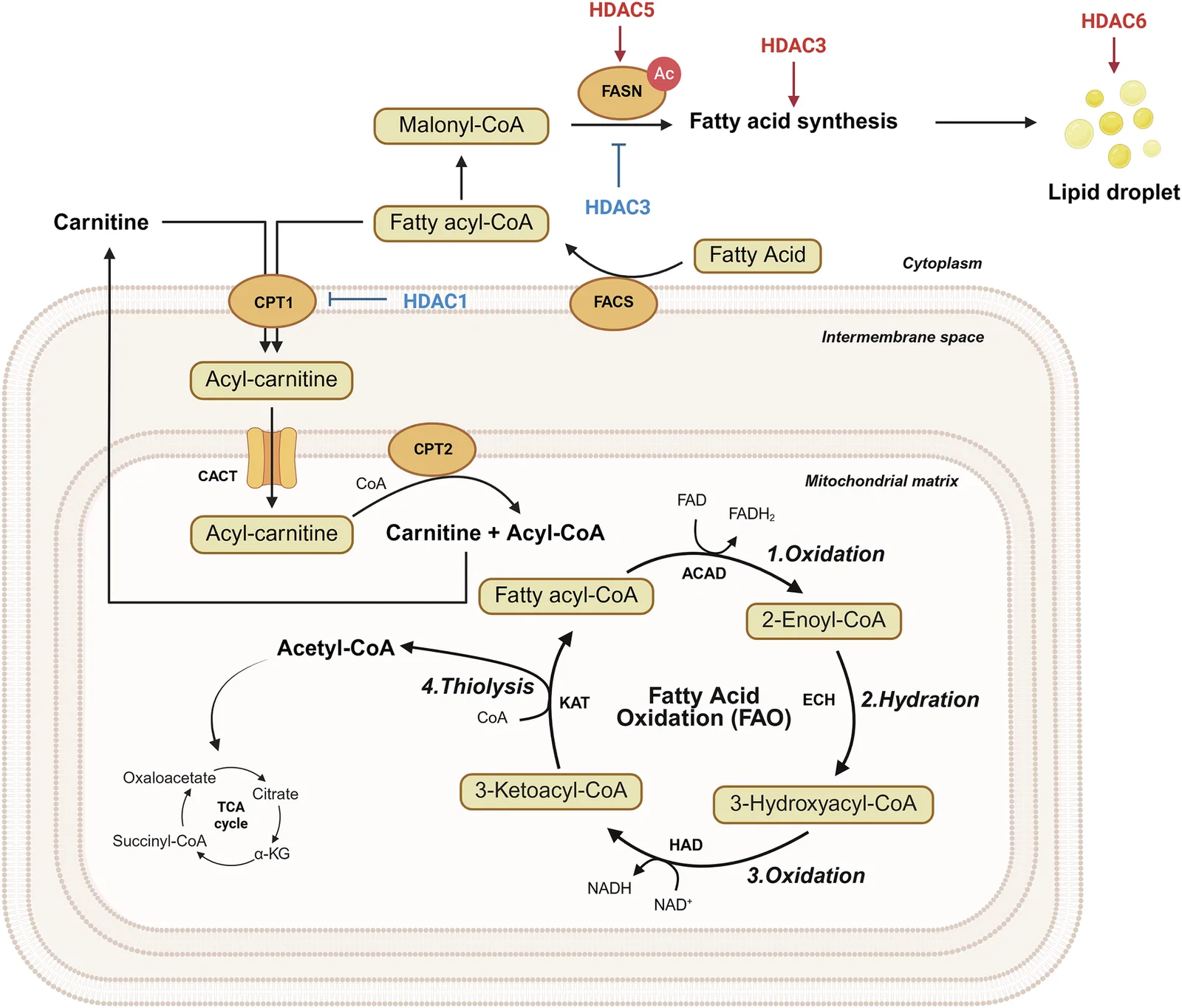
Histone deacetylase regulation of fatty-acid oxidation and lipid synthesis. This schematic depicts fatty-acid catabolism through the mitochondrial β-oxidation pathway and highlights a regulatory role of histone deacetylase 1 (HDAC1) in the regulation of CPT1-dependent fatty-acid entry and subsequent oxidation. Cytosolic fatty acids are activated to fatty acyl-CoA by acyl-CoA synthetases and converted to acyl-carnitine through CPT1 at the outer mitochondrial membrane. Transport across the inner membrane occurs via carnitine–acylcarnitine translocase (CACT), followed by reconversion to fatty acyl-CoA by CPT2 in the matrix, enabling cyclic β-oxidation steps that generate acetyl-CoA, FADH_2_ and NADH. Acetyl-CoA is converted to malonyl-CoA by acetyl-CoA carboxylase, and fatty-acid chains are synthesized by FASN, with HDAC3, HDAC5 and HDAC6 being key regulatory steps. TCA, tricarboxylic acid.

TNBC exhibits reduced expression of the epithelial transcription factor ovo-like 2 (OVOL2)^83^. Loss of OVOL2 promotes FAO, supporting cancer stemness and tumour initiation. Mechanistically, OVOL2 recruits HDAC1 to STAT2-associated transcriptional complex, repressing FAO-related genes, including *CPT1A* and *CPT1B*. These findings establish HDAC1 as a direct epigenetic regulator of FAO in cancer (Fig. 5). Consistently, in colorectal cancer (CRC), nuclear valosin-containing protein (VCP) facilitates tumour progression by binding to HDAC1 and promoting its degradation, thereby upregulating CPT1A expression^84^. Disruption of this regulatory axis by either entinostat-mediated HDAC1/2 inhibition or small interfering RNA-based HDAC1 depletion reduces the expression of FAO-associated genes and inhibits cancer-cell proliferation, thereby implicating HDAC1 as a key driver of malignant progression via FAO control. Furthermore, HDAC1 negatively regulates mitochondrial HMG-CoA synthase (HMGCS2) expression, suppressing ketone body synthesis from acetyl-CoA generated by FAO^85^. This suggests that HDAC1 controls not only the initiation of fatty-acid catabolism but also the downstream metabolic fate of its products, thereby extending its role as a comprehensive epigenetic regulator of FAO. Together, these findings identify HDAC1 as an epigenetic regulator of FAO in cancer through transcriptional control, thereby linking mitochondrial fatty-acid metabolism to tumour progression (Table 1).

### Histone deacetylase control of fatty-acid synthesis

De novo fatty-acid synthesis in mammalian cells is governed by nutrient and hormonal cues that converge on the lipogenic transcription factors SREBP1c and ChREBP, as well as on downstream enzymes such as acetyl-CoA carboxylase (ACC, encoded by *ACACA*) and fatty-acid synthase (FASN)^86^. Emerging evidence indicates that this lipogenic programme is subject to epigenetic regulation, with HDACs modulating transcriptional output at lipogenic gene loci^71^. Through both chromatin-based and non-histone mechanisms, HDACs contribute to the maintenance of oncogenic lipogenesis.

FASN is the terminal enzyme in de novo lipogenesis and plays a crucial role in supporting cell proliferation. FASN activity is regulated by reversible acetylation, with acetylation mediated by the lysine acetyltransferase 8 (KAT8) and deacetylation by HDAC3. HDAC3-dependent deacetylation stabilizes FASN by preventing its proteasomal degradation, thereby sustaining de novo lipogenesis and promoting tumour cell proliferation^87^. In prostate cancer (PCa), Homeobox B13 (HOXB13) is a critical regulator of androgen receptor activity and androgen-dependent PCa growth. HOXB13 recruits HDAC3 to lipogenic enhancers, leading to histone deacetylation and suppression of FASN expression, thereby inhibiting de novo lipogenesis, tumour metastasis, and applying as a therapeutic strategy for HOXB13 low-PCa^88^. Accordingly, inhibition of HDAC3 increases FASN acetylation, reduces lipogenic flux and suppresses cancer-cell growth. Functionally, HDAC3 displays dual roles in lipid metabolism, acting at the post-translational and transcriptional levels through distinct molecular mechanisms. By contrast to its role in stabilizing FASN, HDAC3 recruited by HOXB13 inhibits de novo lipogenesis in PCa. Beyond HDAC3, lipogenic dependency modulates the therapeutic response to HDAC-targeted interventions in cancer. The HDAC5–LSD1 complex promotes de novo lipogenesis by activating the promoter of FASN^89^. Disruption of this complex suppresses lipogenic gene expression, leading to reduced lipid accumulation and impaired proliferation in RCC. Additionally, HDAC6 is overexpressed in CRC and is associated with a poor prognosis. The selective HDAC6 inhibitor ITF3756 reduces CRC cell viability and induces lipid accumulation, and its antitumour effect is enhanced in combination with bortezomib^90^ (Fig. 5). Collectively, these studies position HDACs as central integrators of fatty-acid metabolism and coordinating nutrient availability with chromatin-based transcriptional regulation. Disruption of HDAC-dependent lipogenic control represents a key pathogenic axis linking metabolic dysregulation to metabolic disease and metabolism-driven cancers (Table 1).

## Amino acid metabolism

Amino acid metabolism plays a critical role in tumour development and therapeutic response^91^. Amino acids contribute to tumorigenesis and antitumour immunity by regulating gene transcription, signalling pathways, and nutrient availability. Among them, glutamine is a key metabolic substrate that supports the rapid proliferation of cancer cells by providing both carbon and nitrogen sources for energy production and biosynthesis^92^. Importantly, glutamine and broader amino acid metabolism programmes are tightly regulated by epigenetic modification.

### Histone deacetylases in glutamine metabolism

**Fig. 6:**
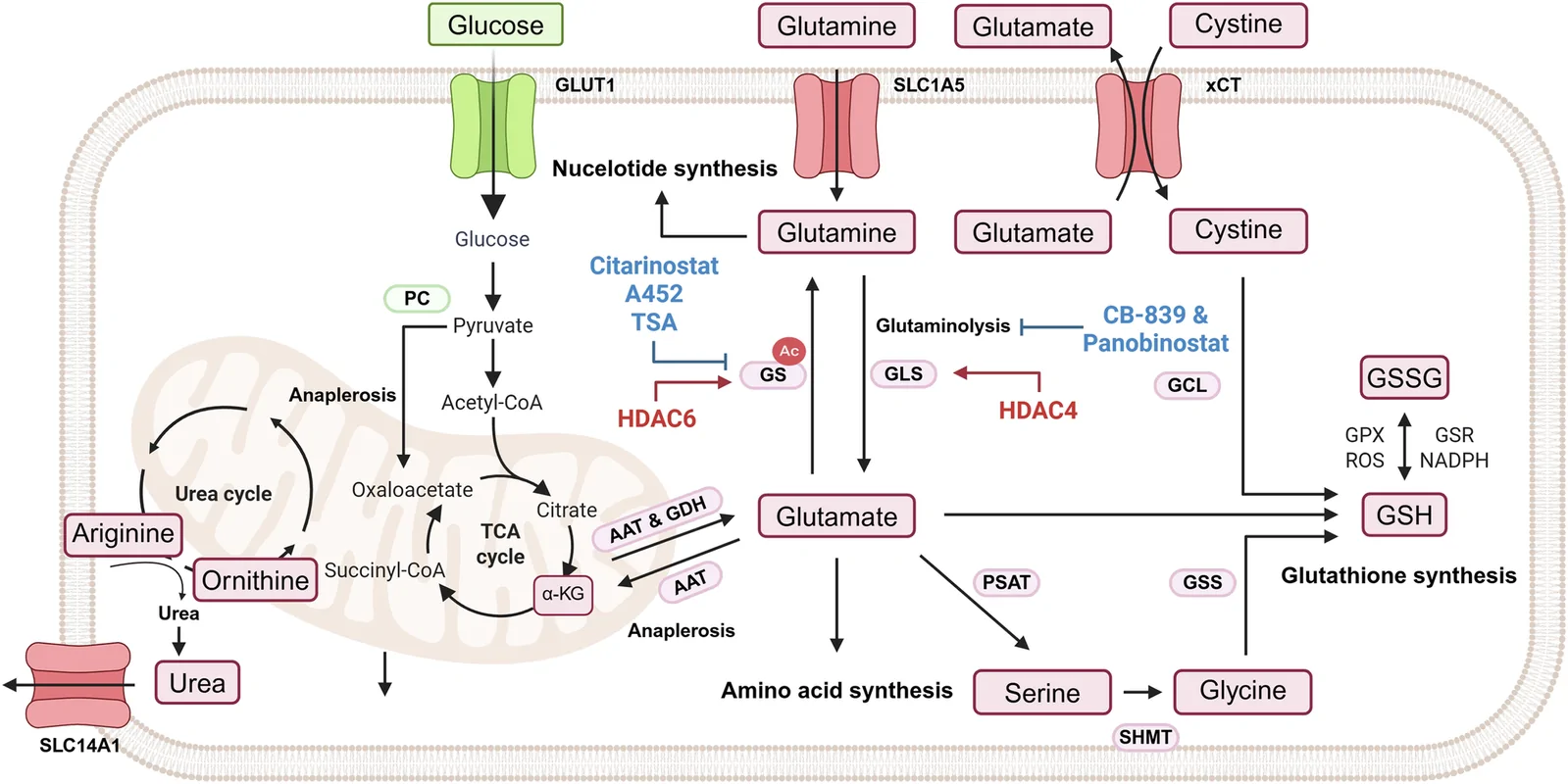
Histone deacetylase-linked glutamine metabolism, anaplerosis, amino acid biosynthesis and glutathione-dependent redox defence. This figure illustrates the glutamine metabolic cycle frequently exploited by cancer cells and highlights histone deacetylase (HDAC)-dependent nodes. Glutamine uptake via SLC1A5 supplies intracellular glutamine, which is converted to glutamate by glutaminase (GLS), supporting glutaminolysis and anaplerotic entry into the tricarboxylic acid (TCA) cycle through α-ketoglutarate-linked reactions. The diagram also depicts diversion of glutamine/glutamate-derived carbon and nitrogen into amino acid synthesis and nucleotide synthesis, supporting proliferative demands. In parallel, the cysteine (xCT) pathway and enzymes such as glutamate-cysteine ligase (GCL), glutathione synthetase (GSS), glutathione peroxidase (GPX), glutathione reductase (GSR) are mapped to glutathione (GSH/GSSG) synthesis and recycling, illustrating antioxidant defences reliant on NADPH. HDAC6 regulates glutamine synthetase (GS)-mediated conversion of glutamate to glutamine, thereby supporting nucleotide synthesis. A combination strategy (CB-839 plus panobinostat) is highlighted to illustrate therapeutic disruption of glutamine metabolism alongside HDAC inhibition, thereby compromising redox homeostasis and metabolic plasticity. ROS, reactive oxygen species.

Glutamine in cancer cells plays an essential role by supplying nitrogen for amino acid and nucleotide synthesis and serving as a carbon source for cellular metabolism through the TCA cycle. Consequently, most cancer cells are highly dependent on glutamine metabolism to sustain their energy demands. Accordingly, stromal cells, including cancer-associated fibroblasts (CAFs), exist in glutamine-deficient environments within tumours. Glutamine starvation suppresses class I HDAC activity, which in turn enhances TGFβ signalling and promotes the conversion to myofibroblastic CAFs^93^. CB-839 (telaglenastat), a glutaminase (GLS) inhibitor, in combination with panobinostat, has a cytotoxic effect in multiple myeloma^94^. In non-small-cell lung cancer (NSCLC), HDAC4 directly interacts with GLS and deacetylates it at Lys311, which reduces GLS interaction with TRIM21, an E3 ubiquitin ligase, thereby preventing K63-linked ubiquitination and enhancing GLS activity to promote glutamine metabolism and tumour progression^95^. These findings suggest that the HDAC4–GLS axis represents a novel post-translational mechanism linking glutamine metabolism to lung-cancer tumorigenesis. Beyond glutamine catabolism, HDACs also regulate glutamine anabolism through modulation of glutamine synthetase (GS). In GBM, where tumour cells frequently encounter nutrient-limited microenvironments, GS-mediated glutamine anabolism is critical for sustaining cell growth and survival under conditions of extracellular glutamine deprivation. HDAC6 directly promotes the deacetylation of GS in glutamine-depleted conditions, which in turn enhances GS protein stability by protecting it from ubiquitin-dependent proteasomal degradation^16^. Through this regulatory mechanism, HDAC6 maintains intracellular glutamine pools, thereby supporting anabolic processes essential for tumour growth. Notably, GS-dependent glutamine synthesis fuels de novo nucleotide biosynthesis. Collectively, these studies demonstrate that HDAC6 regulates glutamine metabolism through post-translational control of key metabolic enzymes, thereby influencing cancer-cell growth and survival (Tables 1 and 2, Fig. 6).

### Histone deacetylases in amino acid metabolism

Arginine is a conditionally essential amino acid that serves as a central node in nitrogen metabolism, nitric oxide synthesis and cancer metabolism reprogramming^96^. Membranous SLC14A1 is a urea transporter that regulates nitrogen metabolism by facilitating urea efflux, the terminal step of amino acid-derived nitrogen disposal^97^. In urothelial carcinoma (UC), SLC14A1 functions as a tumour suppressor, inhibiting cell proliferation and metastasis. However, SLC14A1 expression is frequently downregulated in UC^98^. Inhibition of membranous SLC14A1 prevents urea and arginine accumulation, and nuclear SLC14A1 recruits HDAC1 to inhibit oncometabolite genes, including *HK2* and *delta* *4-desaturase* (*DEGS1)*. Through this mechanism, SLC14A1 constrains both glycolytic and lipid-derived oncometabolite production, thereby suppressing UC progression. It remains unclear whether HDAC-dependent regulatory mechanisms extend to other amino acid pathways, including serine, glycine and leucine, in cancer metabolism reprogramming. Therefore, further studies are warranted to elucidate the role of HDACs in the regulation of these amino acid metabolic pathways.

## Conclusions and future directions

The intricate interplay between metabolism and epigenetics represents a rapidly evolving area in cancer research, offering important insights into how cellular metabolic states influence gene regulation and tumour progression. In this review, we focused on HDAC-mediated regulation of cancer metabolic reprogramming, with particular emphasis on glucose, lipids and amino acid metabolism.

**Fig. 7:**
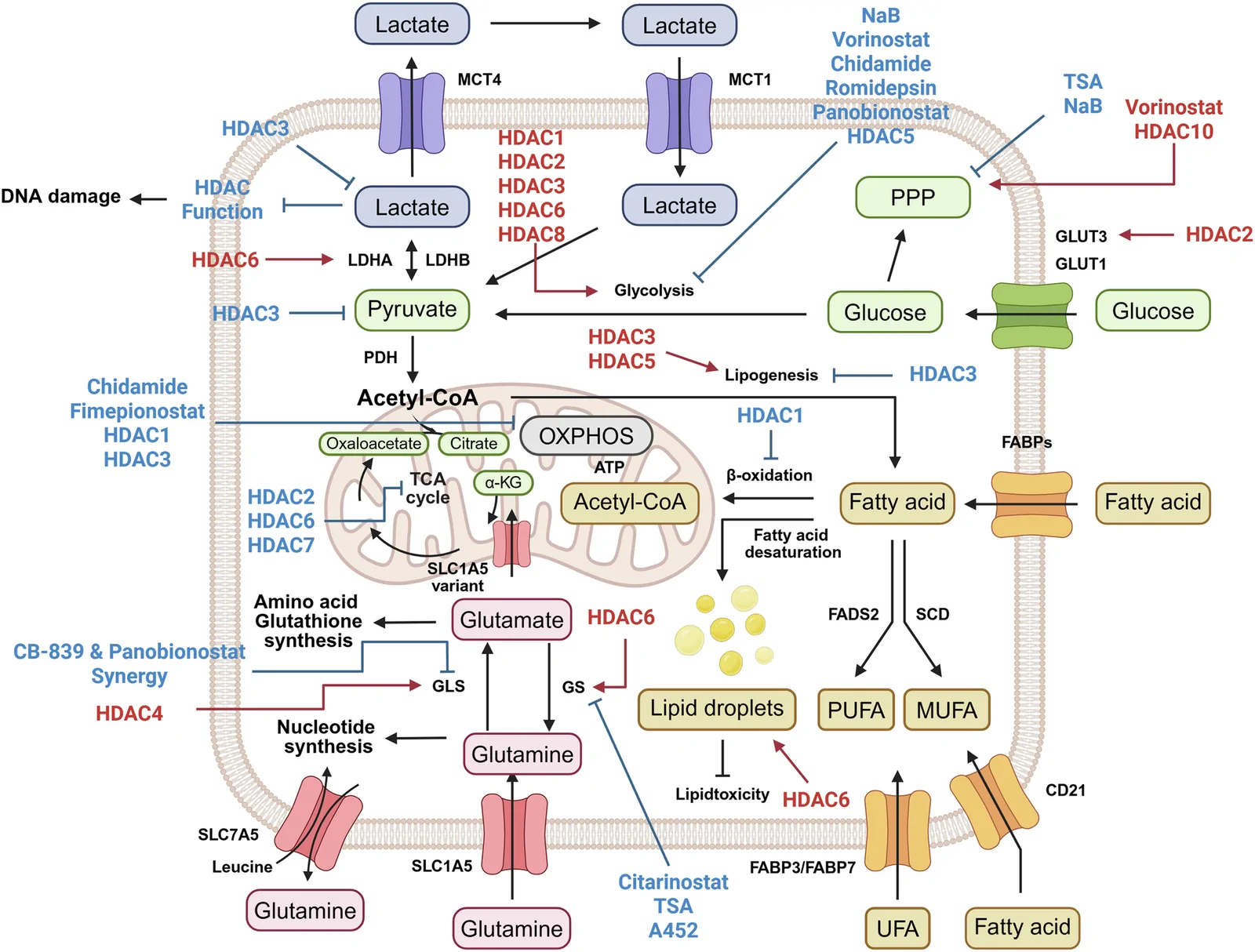
Integrated network of histone deacetylase-driven metabolic reprogramming in cancer. This integrated model summarizes how histone deacetylase (HDAC) isoforms coordinate glucose, lipid and amino acid metabolism in cancer cells, including glycolysis, the pentose phosphate pathway (PPP), the tricarboxylic-acid (TCA) cycle, oxidative phosphorylation (OXPHOS), fatty-acid oxidation (FAO), fatty-acid synthase (FAS) and glutamine metabolism. The network highlights metabolic intermediates that are regulated by HDACs and can be targeted by HDAC inhibitors. MUFA, monounsaturated fatty acid; PUFA, polyunsaturated fatty acid; TSA, trichostatin A; UFA, unsaturated fatty acid.

Across multiple aspects of carbohydrate metabolism, including gluconeogenesis, glycogen metabolism, glycolysis, PPP and mitochondrial metabolism, HDACs exert multi-layered regulatory functions. Through this regulation, HDACs modulate metabolic enzymes (e.g. HK, FBP1, GS), key metabolites and transcription factors such as c-Myc, thereby influencing cellular signalling networks^52,58^ (Fig. 7). Consistent with these observations, numerous studies have demonstrated that HDAC inhibitors disrupt glycolytic metabolism and suppress metastasis and tumour progression. Importantly, the regulatory functions of HDACs extend beyond carbohydrate metabolism to encompass lipid and amino acid metabolic pathways that are essential for tumour growth and adaptation. HDAC1, HDAC3, HDAC5 and HDAC6 are major regulators of lipid metabolism through the regulation of FAO and FAS in cancer and are associated with a poor prognosis. In glutamine metabolism, HDAC6 has been shown to regulate GS activity and stability, thereby supporting cancer-cell survival under metabolic stress^16^. Collectively, these findings indicate that class I HDACs (HDAC1, HDAC2, HDAC3 and HDAC8) and class II HDACs (HDAC5, HDAC6 and HDAC7) play critical roles in cancer-associated metabolic reprogramming (Table 1). Despite extensive research, most studies to date have primarily focused on the broad anticancer effects of pan-HDAC inhibitors rather than on the isoform-specific molecular mechanisms by which individual HDACs regulate metabolic enzymes and discrete steps within metabolic pathways (Table 2). Consequently, although HDAC inhibitors are well documented to suppress cancer-cell proliferation and survival, the therapeutic potential of selectively targeting individual HDAC isoforms remains largely unexplored, particularly in the context of metabolic regulation. Thus, the development of isoform-specific HDAC inhibitors represents a promising strategy for the precise targeting of metabolic dependencies, an approach that will be facilitated by a deeper understanding of isoform-specific HDAC metabolism axes.

Despite growing interest in metabolic epigenetic crosstalk, the roles of protein acetylation and deacetylation in cancer metabolic reprogramming remain incompletely understood. HATs and HDACs are frequently dysregulated in cancer and modulate oncogenic signalling and tumour progression, thereby reshaping gene-expression programmes and metabolic phenotypes. Notably, HDACs have emerged as key epigenetic regulators in cancer biology, with their aberrant expression or activity directly linked to the silencing of tumour-suppressor genes, dysregulation of metabolic checkpoints and promotion of metabolic adaptations that support cancer-cell survival and proliferation. In addition, cancer metabolic reprogramming leads to lactate accumulation and promotes protein lactylation as a post-translational modification, with emerging evidence identifying several HDACs as deacetylases. However, how acetylation and lactylation dynamics directly regulate metabolic enzymes and contribute to cancer metabolic reprogramming remains largely undefined.

Although the present review focuses on the class-specific roles of HDACs in cancer metabolic regulation, this scope itself represents an important limitation. A truly comprehensive understanding of epigenetically driven metabolic reprogramming in cancer will require the integration of other epigenetic regulators beyond HDACs, including HATs, histone methyltransferases and demethylases, and chromatin remodelling complexes. However, studies simultaneously examining multiple epigenetic layers in the context of cancer metabolism remain limited, and the field has yet to establish a unified framework that captures their collective contributions. As such, future research systematically integrating these regulators will be essential. Even within the scope of HDAC research itself, several important limitations must be acknowledged. Our mechanistic understanding remains constrained, largely due to the limitations of current experimental approaches. Most studies rely on broad-spectrum HDAC inhibitors, which have hindered the elucidation of molecular mechanisms attributable to specific isoforms. The off-target effects of these inhibitors further complicate mechanistic interpretation and must be carefully considered. Additionally, further clinical data are needed to better establish the relationship between individual HDAC isoforms and cancer metabolism in human-disease contexts. Collectively, addressing these limitations and advancing our understanding of the interplay between HDAC-mediated and broader epigenetic regulation of metabolic enzymes will provide critical insights into cancer metabolic reprogramming and may ultimately uncover novel therapeutic targets and strategies.
